# Supplementary material for: Tracing day-zero and forecasting the COVID-19 outbreak in Lombardy, Italy: A compartmental modelling and numerical optimization approach
Source: PLoS One. 2020 Oct 30;15(10):e0240649. doi: 10.1371/journal.pone.0240649 (PMC7598513; doi:10.1371/journal.pone.0240649)
Supplement: S1 File — (PDF) [file pone.0240649.s001.pdf]

S1 File

Data

All the relevant data used in this paper are publicly available and accessible at http://www.salute.gov.it/. The reported cumulative numbers of cases from February 21 to March 19 are listed in S1 Table . The reported numbers of confirmed cases from March 20 to May 20 are listed in S2 Table .

S1 **Table.** Reported cumulative numbers of cases for Lombardy, Italy (February 21-March 19)

Calibration Data				Validation Data			
Date	Infected	Recovered	Deaths	Date	Infected	Recovered	Deaths
Feb 21	15	0	0	March 09	5469	646	333
22	54	0	1	10	5791	896	468
23	1101	0	6	11	7280	900	617
24	172	0	9	12	8725	1085	744
25	240	0	9	13	9820	1198	880
26	258	0	9	14	11685	1660	966
27	403	40	14	15	13272	2011	1218
28	531	40	17	16	14649	2368	1420
29	615	40	23	17	16220	2485	1640
March 01	984	73	31	18	17713	3488	1959
02	1254	139	38	19	19884	3778	2168
03	1529	139	55				
04	1820	250	73				
05	2251	376	98				
06	2612	469	135				
07	3420	524	154				
08	4189	550	267				

S2 **Table.** Reported numbers of confirmed cases for Lombardy, Italy (March 20-May 4)

Date	Infected	Recovered	Deaths	Date	Infected	Recovered	Deaths
March 20	15420	4295	2549	April 16	33090	18396	11608
21	17370	5050	3095	17	33434	18850	11851
22	17885	5865	3456	18	34195	19136	12050
23	18910	6075	3776	19	34497	19526	12213
24	19868	6657	4178	20	34587	20008	12376
25	20591	7281	4474	21	33978	21374	12579
26	22189	7839	4861	22	34242	22110	12740
27	23895	8001	5402	23	33873	23352	12940
28	24509	8962	5944	24	34368	23782	13106
29	25392	9255	6360	25	34473	24227	13269
30	25006	10337	6818	26	35166	24398	13325
31	25124	10885	7199	27	35441	24589	13449
April 1	25765	11415	7593	28	35744	25029	13575
02	25876	12229	7960	29	36122	25333	13679
03	26189	13020	8311	30	36211	25749	13772
04	27220	13242	8656	May 1	36473	26136	13860
05	28124	13426	8905	2	36667	26146	14189
06	28469	13863	9202	3	36926	26371	14231
07	28343	14498	9484	4	37307	26504	14294
08	28545	15147	9722				
09	29074	15706	10022				
10	29530	16280	10238				
11	30258	16823	10511				
12	31265	17166	10621				
13	31935	17478	10901				
14	32363	17821	11142				
15	32921	17855	11377				

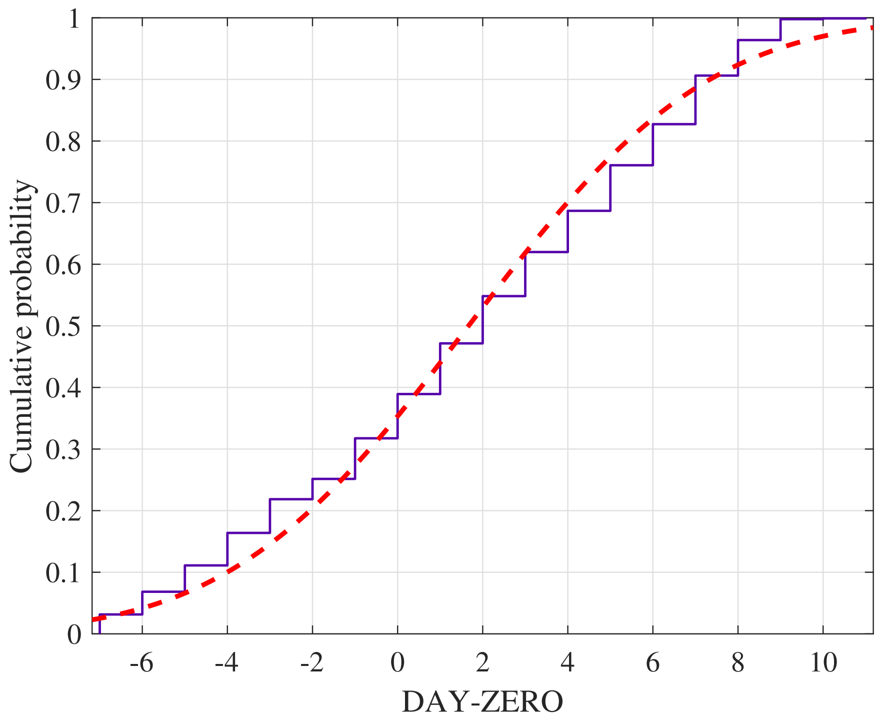

S1 Fig. Cumulative probability distribution of the optimal values of day-zero as obtained by running the optimization problem using a grid of $20 \times 13 \times 15$ initial guesses, thus using a 2 days step for the day-zero within the interval 27 December 2019-5th of February 2020 i.e. ± 20 days around the 16th of January, a step of 0.05 within the interval (0.3, 0.9) for β and a step of 0.02 within the interval (0.01, 0.29) for $\epsilon =$. The best fit was obtained with the Normal CDF with $\mu = 1.67$ (95% CI: 1.53, 1.80) (thus corresponding to the 14th of January) and $\sigma = 4.43$ (95% CI:4.33, 4.52).

Fitting the Distributions of the Optimal Values

We fitted the cumulative probability distributions of day-zero β, ϵ using several functions including the Normal, Log-normal, Weibull, Beta, Gamma, Burr, Exponential and Birnbaum-Saunders CDFs and kept the one resulting in the maximum Log-likelihood. For the day-zero, the best fit to the distribution of the optimal values was obtained by the Normal CDF:

$$F(x|\mu,\sigma) = \frac{1}{2}(1 + erf(\frac{x-\mu}{\sigma\sqrt{2}})),$$

with mean value $\mu = 1.67$ (95% CI: 1.53, 1.80) and $\sigma = 4.43$ (95% CI:4.33, 4.52).

The cumulative distribution of day-zero and the resulting exponential distribution are given in S1 Fig.

For the distribution of β values the best fitting distribution was obtained by the Burr CDF which is a three-parameter family of curves given by:

$$F(x|\alpha,c,k) = 1 - \frac{1}{(1 + (\frac{x}{\alpha})^c)^k}, x, \alpha, k, c > 0$$

with $\alpha = 0.654$ (95% CI: 0.653, 0.655), $c = 276.061$ (95% CI: 248.911, 306.172), $k = 0.0537$ (95% CI: 0.048, 0.060). Thus, the resulting mean value is $\beta = 0.70$. The

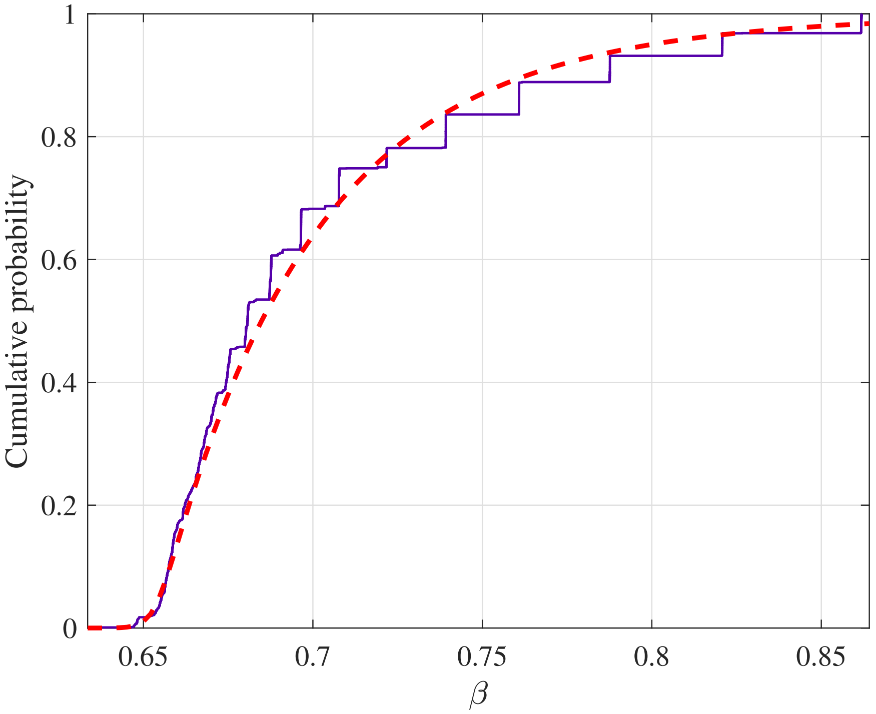

S2 Fig. Cumulative probability distribution of the optimal values of β as obtained by running the optimization problem using a grid of $20 \times 13 \times 15$ initial guesses, thus using a 2days step for the day-zero within the interval 27 December 2019-5th of February 2020 i.e. ± 20 days around the 16th of January, a step of 0.05 within the interval (0.3, 0.9) for β and a step of 0.02 within the interval (0.01, 0.29) for ϵ. The best fit was obtained with the Burr CDF, with $\alpha = 0.654$ (95% CI: 0.653, 0.655), $c = 276.061$ (95% CI: 248.911, 306.172), $k = 0.0537$ (95% CI: 0.048, 0.060).

cumulative distribution of *beta* and the resulting Burr CDF distribution are given in S2 Fig.

For ϵ, the best fit to the distribution of the optimal values was obtained by a Birnbaum-Saunders CDF:

$$F(x|\mu, \alpha) = \Phi(\frac{1}{\alpha}(\sqrt{\frac{x}{\mu}} - \sqrt{\frac{\mu}{x}}),$$

with $\mu = 0.0492$ (95% CI: 0.048, 0.0505) (scale parameter) and $\alpha = 0.934$ (95% CI: 0.914, 0.954) (shape parameter). $\Phi(x)$ denotes the standard normal CDF. The mean value is given by:

$$\mu(1 + \frac{\alpha^2}{2})$$

Thus, the mean value is given by $\epsilon = 0.0707$.

The cumulative distribution of ϵ and the resulting exponential distribution are given in S3 Fig.

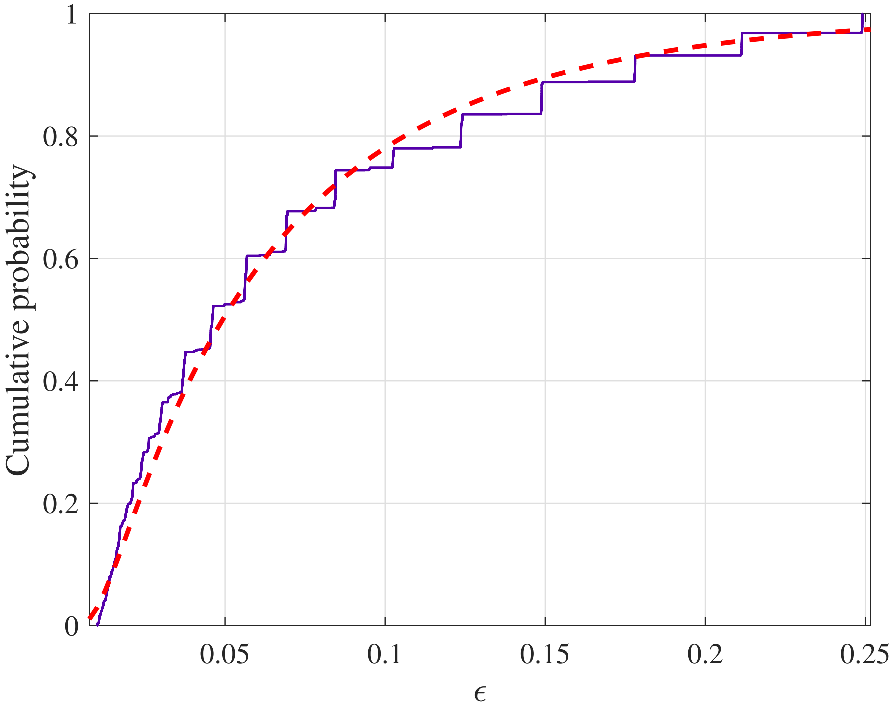

S3 Fig. Cumulative probability distribution of the optimal values of ϵ as obtained by running the optimization problem using a grid of $20 \times 13 \times 15$ initial guesses, thus using a 2days step for the day-zero within the interval 27 December 2019-5th of February 2020 i.e. ± 20 days around the 16th of January, a step of 0.05 within the interval (0.3, 0.9) for β and a step of 0.02 within the interval (0.01, 0.29) for ϵ. The best fit was obtained by a Birnbaum-Saunders CDF, $\mu = 0.0492$ (95% CI: 0.048, 0.0505) (scale parameter) and $\alpha = 0.934$ (95% CI: 0.914, 0.954) (shape parameter).
